# Direct visualization of phase-matched efficient second harmonic and broadband sum frequency generation in hybrid plasmonic nanostructures

**DOI:** 10.1038/s41377-020-00414-4

**Published:** 2020-10-22

**Authors:** Zhe Li, Brian Corbett, Agnieszka Gocalinska, Emanuele Pelucchi, Wen Chen, Kevin. M. Ryan, Pritam Khan, Christophe Silien, Hongxing Xu, Ning Liu

**Affiliations:** 1 Department of Physics and Bernal Institute, University of Limerick, Limerick, Ireland; 2 The School of Physics and Technology, Institute for Advanced Studies and Center for Nanoscience and Nanotechnology, Wuhan University, Wuhan, 430072 China; 3 Tyndall National Institute, University College Cork, Cork, Ireland; 4 Institute of Physics, École Polytechnique Fédérale de Lausanne (EPFL), CH, 1015 Lausanne, Switzerland; 5 Department of Chemical Sciences and Bernal Institute, University of Limerick, Limerick, Ireland

**Keywords:** Nanowires, Photonic devices

## Abstract

Second harmonic generation and sum frequency generation (SHG and SFG) provide effective means to realize coherent light at desired frequencies when lasing is not easily achievable. They have found applications from sensing to quantum optics and are of particular interest for integrated photonics at communication wavelengths. Decreasing the footprints of nonlinear components while maintaining their high up-conversion efficiency remains a challenge in the miniaturization of integrated photonics. Here we explore lithographically defined AlGaInP nano(micro)structures/Al_2_O_3_/Ag as a versatile platform to achieve efficient SHG/SFG in both waveguide and resonant cavity configurations in both narrow- and broadband infrared (IR) wavelength regimes (1300–1600 nm). The effective excitation of highly confined hybrid plasmonic modes at fundamental wavelengths allows efficient SHG/SFG to be achieved in a waveguide of a cross-section of 113 nm × 250 nm, with a mode area on the deep subwavelength scale (*λ*^2^/135) at fundamental wavelengths. Remarkably, we demonstrate direct visualization of SHG/SFG phase-matching evolution in the waveguides. This together with mode analysis highlights the origin of the improved SHG/SFG efficiency. We also demonstrate strongly enhanced SFG with a broadband IR source by exploiting multiple coherent SFG processes on 1 µm diameter AlGaInP disks/Al_2_O_3_/Ag with a conversion efficiency of 14.8% MW^−1^ which is five times the SHG value using the narrowband IR source. In both configurations, the hybrid plasmonic structures exhibit >1000 enhancement in the nonlinear conversion efficiency compared to their photonic counterparts. Our results manifest the potential of developing such nanoscale hybrid plasmonic devices for state-of-the-art on-chip nonlinear optics applications.

## Nonlinear optics: plasmonic nanostructures

Plasmonic waveguides and microdisk cavities can provide an efficient means of nonlinear wavelength conversion of light in a chip-compatible platform. Zhe Li and coworkers from Ireland, China and Switzerland fabricated nano- and micro-scale waveguides and cavities from the compound semiconductor AlGaInP, which were then transferred to plasmonic substrates composed of SiO_2_/Si coated with thin layers of metal oxide (Al_2_O_3_) and silver (Ag). When pumped with near-infrared light in the 1300 to 1600 nm region, efficient second harmonic generation (SHG) and sum frequency generation (SFG) were observed with a conversion efficiency of up to 14.8% MW^−1^. Control experiments with the same structures on plain glass substrates indicate that the plasmonic versions offer >1000 times enhancement in the nonlinear conversion efficiency. The results suggest a promising future for integrated optics employing plasmonics.

## Introduction

The development of broadband and efficient optical frequency up-converters integrated with nanophotonics systems is highly desired for a range of applications, including biosensing^1^, imaging^2,3^, and photonic circuitry^4–6^. These devices can be realized by second harmonic generation (SHG), the process in which two low-energy photons of the same frequency are converted into one high-energy photon, and sum frequency generation (SFG), the process in which two low-energy photons of different frequencies are converted into one high-energy photon by exploiting the nonzero second-order susceptibility tensor of noncentrosymmetric materials. As these processes are inherently weak, improvement of their conversion efficiency is predominantly controlled by enhancing the nonlinear light–matter interactions, which is associated with light confinement^7^, spatial overlap^8^, and phase matching^9^ of electromagnetic fields at fundamental and nonlinear frequencies. To fulfill these requirements, various complex microstructures composed of single or multiple nonlinear materials have been fabricated, demonstrating high conversion efficiencies in photonic devices such as waveguides^9^ and microring resonators^10,11^. However, further minimization of these devices is ultimately limited by the diffraction limit of light. In contrast, metallic nanostructures enable extremely small mode volumes through surface plasmon resonances^12,13^, whereas the SHG/SFG conversion efficiency is hampered by the naturally small nonlinear coefficient of metals^8,14–16^. A promising way to overcome these limitations is to exploit hybrid plasmonic systems, which integrate a metal with nonlinear nanomaterials and allow effective interplay between the subwavelength light confinement and large nonlinear susceptibility^17–21^.

Two methods are commonly used to achieve strong SHG. One is to exploit localized resonances for mode matching at fundamental and SHG wavelengths to enhance the conversion^7,22^, which is often used to realize a high conversion efficiency/volume ratio. To achieve a high total conversion efficiency and/or compatibility with integrated photonics, a waveguide configuration is applied^23^, where a phase-matching condition is often needed when the fundamental wave propagates over a distance of a few wavelengths^9,24^.

Noncentrosymmetric compound III–V semiconductors, such as GaInP and GaAs, are well known for their strong second-order nonlinear effects^25–29^ and have well-developed top-down fabrication procedures, compatible with further large-scale on-chip integration. For nonlinear III–V semiconductor nanostructures in close vicinity to a metal, strong and controllable SHG is expected. However, realistic semiconductor materials possess a nonlinear dispersion relationship (*k*_2__ω_ ≠ 2*k*_ω_)^30,31^. For surface plasmon mode propagation at the semiconductor–metal interface, the difference between *k*_2__ω_ and 2*k*_ω_ is further widened^12^. In other words, the coherent length of SHG in semiconductor-metal waveguides is extremely short, usually within a few micrometers. For this reason, although several hybrid plasmonic waveguides have been reported for SHG applications in the literature^7,17,23,32^, phase matching in hybrid plasmonic waveguides has yet to be demonstrated/optimized^7,23,32^.

Here, by exciting the highly confined transverse magnetic (TM) hybrid plasmonic modes at the fundamental frequencies, we have achieved effective up-conversion of light from communication wavelengths to the red part of the spectrum using SHG and SFG in lithographically defined 110 nm-thick AlGaInP-based waveguides and resonant cavities/Al_2_O_3_(6 nm)/Ag^33^. We show that efficient SHG/SFG (>1500 times that of bare waveguides on glass) can be realized in hybrid plasmonic waveguides via effective phase matching enabled by the excitation of higher-order modes in the waveguides. Remarkably, direct far-field visualization of the phase-matching evolution is achieved in our devices. By employing the strong electric field confinement and enhancement offered by the hybrid plasmonic modes, efficient SHG and SFG (>14% W^−1^ cm^−2^) are realized in waveguides with a physical cross-sectional area of *λ*^2^/16, exhibiting a deep subwavelength mode area of *λ*^2^/135 at the fundamental wavelength (FW) of 1340 nm.

To further demonstrate the advantage of our approach for broadband up-conversion, the waveguides are reduced to 1 μm disks. By exploiting multiple coherent SFG processes arising from the broadband IR source, an SFG conversion efficiency as high as 14.8% MW^−1^ is reached, which is five times that offered by the narrowband IR source and is comparable to the best efficiencies in previous plasmonic nanocavities^8,34^, optical microcavities^28,29^, and hybrid cavities^20^, given the same signal/volume ratio. These results illustrate the significant advantages of hybrid plasmonic structures as a means to achieve highly efficient nonlinear conversion, thereby enabling the miniaturization of integrated photonics.

## Results

To reduce the propagation loss inherent to plasmonic nanostructures, an air-semiconductor-insulator-metal hybrid plasmonic geometry is applied^35^. The 110 nm-thick AlGaInP multilayer ((Al_0.3_Ga_0.7_)_0.5_In_0.5_P/Ga_0.5_In_0.5_P/(Al_0.3_Ga_0.7_)_0.5_In_0.5_P) nanostructures, such as waveguides with rectangular cross-sections and disks, are fabricated and released onto Al_2_O_3_ (6 nm)/Ag (70 nm)/SiO_2_/Si substrates using the methods detailed in ref. ^33^.

### Direct visualization of SHG propagation and amplification in waveguides

**Fig. 1:**
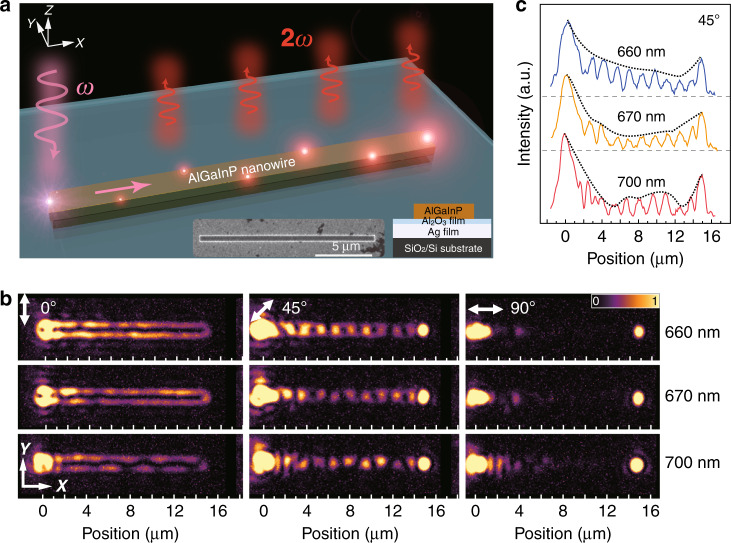
Wavelength- and polarization-dependent characterization of SHG in an AlGaInP waveguide (113 nm × 584 nm × 15 µm)/Al_2_O_3_(6 nm)/Ag(70 nm)/SiO_2_/Si structure. **a** Diagram showing the experimental setup of waveguided SHG. The excitation beam was edge-coupled into the waveguide from the left end. The inset shows an SEM image of the 15 µm waveguide and the schematic of the cross-section. The scale bar is 5 µm. **b** SHG images at three input polarizations and various wavelengths. **c** SHG power vs. position (*x* direction) plots obtained by integrating the SHG intensity along the y direction using images shown in **b** at 660, 670, and 700 nm with an input polarization of 45°

**Fig. 2:**
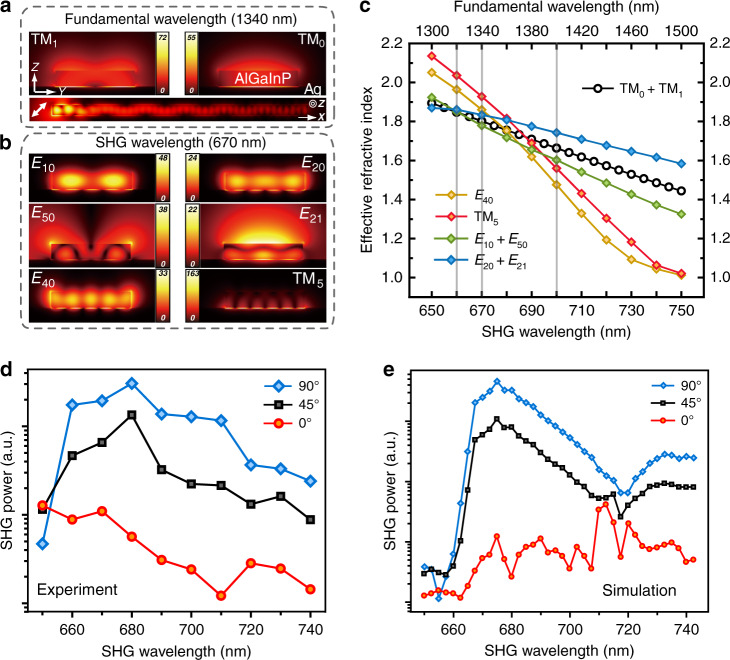
Mode analyses of the waveguided SHG and comparison of the SHG signals obtained at the output end of the AlGaInP waveguide. **a** Numerical simulations of the electric field distribution for the different modes at 1340 nm in the hybrid system and the electric field amplitude |E| distribution on the *xz* plane when the input beam is polarized along 45° with respect to the *x* direction. **b** Numerical simulations of the electric field distribution for the different modes at 670 nm. **c** Simulated effective refractive index of the modes or their combination in **a** and **b** at various wavelengths. **d**, **e** SHG signal obtained at the output end of the AlGaInP waveguide (113 nm × 584 nm × 15 µm)/Al_2_O_3_(6 nm)/Ag(70 nm)/SiO_2_/Si structure at the same input power, different SHG wavelengths, and 0°, 45°, and 90° input polarizations by experiments (**d**) and simulation (**e**)

The SHG of waveguides is measured first. Figure 1 shows the polarization-dependent SHG through a 113 nm × 584 nm × 15 µm (thickness × width × length) waveguide at three different FWs. In this configuration, the fundamental beam is edge-coupled into the waveguide (Fig. 1a)^23,36^ from the left side (see “Methods” and Fig. S1 for details). When input light is focused at the end of a waveguide, scattering of free-space photons at points of structural symmetry breaking (the edge) allows for satisfaction of momentum conservation and therefore excitation of the hybrid plasmonic TM modes in the waveguide^12,36^. Depending on the input polarization, TM_0_, TM_1_, or a combination of both modes is excited at the FW. As shown in Fig. 2a, displaying the simulations by COMSOL multiphysics simulation package of the mode distribution of TM_0_ and TM_1_ (see section 2 of the Supporting Information), these hybrid plasmonic modes are tightly confined to the semiconductor-insulator-metal interfaces, with over 60% of the electric energy contained in the semiconductor region, allowing effective light–matter interactions. To reduce the overlap of SHG wavelengths with the photoluminescence band of AlGaInP (see Fig. S2 of the Supporting Information), we limit the FW within the range of 1300–1600 nm. For the waveguide of 584 nm in width, at a given SHG wavelength, multiple modes are supported. Depending on the effective refractive index of the SHG and that of the fundamental modes, phase-matching conditions are satisfied at specific wavelengths and at certain combinations of modes. Once the SHG efficiency exceeds the propagation losses of the modes, coherent amplification of SHG along the propagation direction is realized at certain wavelengths.

This effect is experimentally observed in the far field, e.g., with an incident beam polarized at 45° with respect to the waveguide, as shown in Fig. 1b. Here, the observed patterns in the far field are attributed to a combination of the TM_0_ and TM_1_ modes at the FW (Fig. 2a)^37^. The periodicity of the obtained patterns is found to be insensitive to the excitation wavelength (Fig. 1b and see Supporting Information section 3 for details). Figure 2c shows the simulated effective refractive index of combination modes of TM_0_ and TM_1_ at different excitation wavelengths. Although multiple modes are supported by the waveguide at a given SHG wavelength, only the modes or combinations of modes with a refractive index closely matching that at the FW are effectively generated. Four SHG modes/combinations of modes are identified (Fig. 2b), which contribute to the SHG signals generated at 45° input polarization. Their effective refractive indices are also plotted in Fig. 2c, which correspond to the E_40_, TM_5_, E_10_ + E_50_, and E_20_ + E_21_ modes. The far-field observation is further enhanced by the excitation of the higher-order low-effective-refractive-index E_50_ and E_21_ modes at the SHG wavelength, which can easily couple to the far field. These leaky modes serve as reporters of the conversion efficiency of the SHG signal along the propagation direction of the waves.

Depending on the propagation loss of the SHG modes and their phase-matching condition to the fundamental TM_0_ and TM_1_ modes, the envelope amplitude of SHG along the waveguide varies^38,39^. Figure 1c shows the line profiles of the SHG signal obtained from the far field as a function of the propagation distance at wavelengths of 660, 670, and 700 nm at 45° input polarization. At 670 nm, after the initial decrease in the SHG signal caused by edge scattering, the SHG intensity increases monotonically along the waveguide because of the satisfaction of the phase-matching condition and the relatively low propagation loss. In contrast, as 660 nm lies within the absorption band of AlGaInP, the propagation loss of the modes is the highest among the three wavelengths. Consequently, the output SHG decreases monotonically as the modes propagate along the waveguide. At 700 nm, the propagation loss is the lowest, but the phase-matching condition is not strictly satisfied (*n*_2ω_ ≠ *n*_ω_). As shown in Fig. 1c, the intensity of the envelope increases up to a distance of 10 µm and decreases afterwards, with the minimum occurring at ~12 µm. From this, we can estimate the coherence length (2*π*/|*k*_2ω_ − 2*k*_ω_|) of the SHG to be 12 µm, which corresponds to |*n*_2ω_ − *n*_ω_| = 0.06 at 700 nm, consistent with the dispersion relationship shown in Fig. 2c.

Given the propagation loss constants in both the fundamental and SHG modes, under the assumption that the SHG signal is much smaller than that of the input fundamental mode and perfect phase matching is satisfied, such as the SHG in the 670 nm case, the variation in the SHG intensity along the long axis of the waveguide can be quantitatively determined by^38^ (see Supporting Information section 4 for details):

1 $$I_{\mathrm {SHG}}\left( x \right) \propto ({{e}}^{ - 2\alpha _1x} - {{e}}^{ - \alpha _2x})^2$$

where *I*_SHG_ is the intensity of the SHG signal, and *α*_1_ and *α*_2_ are the loss constants of the optical modes at the fundamental and SHG wavelengths, respectively. The maximum $I_{\mathrm {SHG}}$ occurs at position:

2 $$x = \ln \left( {\frac{{\alpha _2}}{{2\alpha _1}}} \right)/(\alpha _2 - 2\alpha _1)$$

For SHG at 670 nm, the simulated values are $\alpha _{2,\mathrm {TM}_5} = 2946 {\mathrm {cm}}^{ - 1}$, $\alpha _{\mathrm {2,E}_{40}} = 1042 {\mathrm {cm}}^{ - 1}$, $\alpha _{\mathrm {2,E}_{20}} = 771 {\mathrm {cm}}^{ - 1}$, $\alpha _{\mathrm {2,E}_{10}} = 675 {\mathrm {cm}}^{ - 1}$, $\alpha _{\mathrm {2,E}_{50}} = 700 {\mathrm {cm}}^{ - 1},\alpha _{\mathrm {2,E}_{21}} = 875 {\mathrm {cm}}^{ - 1},\alpha _{\mathrm {1,TM}_0} = 726 {\mathrm {cm}}^{ - 1}$, and $\alpha _{\mathrm {1,TM}_1} = 706 {\mathrm {cm}}^{ - 1}$. Except for the TM_5_ mode, all the modes have a maximum amplification length longer than 10 µm (see Supporting Information Fig. S4a), consistent with our observation at 45° input polarization.

There are, however, other optical modes that satisfy phase-matching conditions but cannot be easily observed in the far field in the current setup. For example, for input light polarized along the long axis of the waveguide, only the TM_0_ fundamental mode is excited at the FW and the phase-matched SHG modes have a higher effective refractive index. Under the current collection geometry, the SHG signal can only be effectively detected in the far field at the end of the waveguide as scattered photons (Fig. 1b).

Figure 2d shows the SHG signals obtained at the output end of the waveguide at different SHG wavelengths and at 0°, 45°, and 90° input polarizations. It is clear from the plots that the output SHG intensity sensitively depends on both the polarization and wavelength. At 90° polarization, the output SHG intensity is the highest, attributed to the highest input coupling efficiency at the FW (see Supporting Information section 5). Figure 2e gives the SHG output efficiency simulated by COMSOL, which yields reasonable agreement with the experimental results. In the hybrid plasmonic waveguide case, even when the phase-matching condition is satisfied, the maximum amplification distance strongly depends on the propagation losses of the fundamental and SHG modes (Eq. (1)). In the 90° polarization case, the phased-matched SHG modes have higher losses, with *α*_2_ ranging from 1042 to 2946 cm^−1^, yielding the maximum amplification length of 4–7 µm. In this waveguide configuration, the highest waveguided SHG is experimentally obtained at 670 nm for a 113 nm × 584 nm × 8 µm waveguide, with a time-averaged 0.36 nW waveguided SHG signal obtained in the far field from a 7.6 mW input power at 1340 nm and a conversion efficiency $\eta _{\mathrm {SH,wg}}$ that is defined as:

3 $$\eta _{\mathrm {SH,wg}} = \frac{{P_{\mathrm {pk - SH,wg}}}}{{P_{\mathrm {pk - FW,wg}}^2L^2}}$$

of 12%${\mathrm {W}}^{ - 1}{\mathrm {cm}}^{ - 2}$, where $P_{\mathrm {pk - FW,wg}}$ is the peak power of edge-coupled input light, $P_{\mathrm {pk - SH,wg}}$ is the peak power of the output SHG signal and *L* is the length of the waveguide. With a shorter waveguide of 4 µm, the conversion efficiency $\eta _{\mathrm {SH,wg}}$ is expected to exceed 96%${\mathrm {W}}^{ - 1}{\mathrm {cm}}^{ - 2}$, matching one of the highest values achieved for lithium niobate photonic waveguides^40^ of larger dimensions.

### Broadband SFG propagation and amplification in waveguides

Limited by the strict phase-matching conditions of SHG, it is challenging to fabricate a photonic nanowaveguide allowing highly efficient, broadband IR-to-visible up-conversion. In our configuration, however, due to the presence of the metal film, more modes are available at each wavelength to interact with each other at the same/different wavelengths to achieve high conversion efficiency through broadband SFG.

**Fig. 3:**
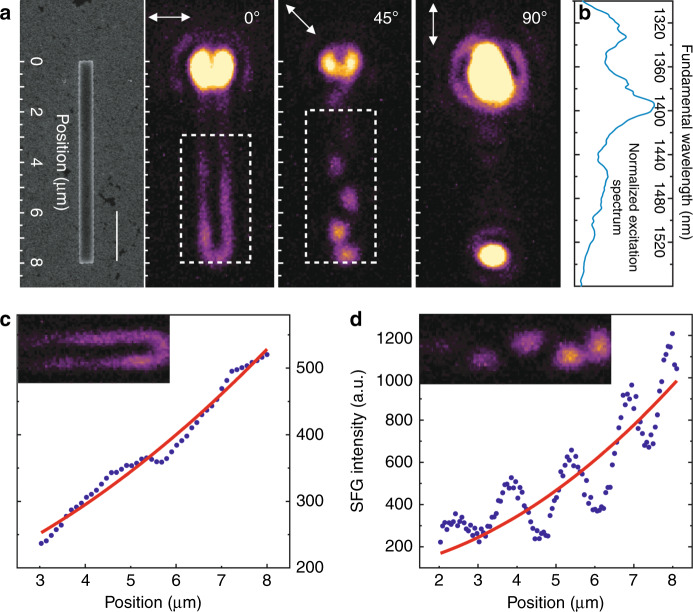
Broadband SFG and characterization of the AlGaInP waveguide (113 nm × 584 nm × 8 µm)/Al_2_O_3_(6 nm)/Ag(70 nm)/SiO_2_/Si structure. **a** SEM and SFG images of the waveguide at various polarization directions. The scale bar indicates 2 µm. **b** Normalized broadband excitation spectrum. **c**, **d** SFG power vs. position (*x* direction) plots obtained by integrating the SFG intensity along the *y* direction at 0° and 45° input polarizations (dashed curves), and the fits (solid curves) to Eq. (1)

Once a super continuum broadband IR source is used as the excitation source (spectrum shown in Fig. 3b), waveguided SFG is generated. In the broadband case, both SHG and SFG are present (diagram shown in Fig. S5). In the following text, we label the up-conversion processes using the broadband excitation light source with the general term SFG. An example of SFG by a 113 nm × 584 nm × 8 µm waveguide is shown in Fig. 3 (SFG spectrum shown in Fig. S6). Similar to SHG, the SFG patterns are clearly observable in the far field at 0° and 45° input polarizations. Furthermore, the SFG signal increases along the long axis of the waveguide much faster than SHG, suggesting different propagation losses at the fundamental and SFG wavelengths. Assuming a similar $\alpha _{\mathrm {FW}} = 720 {\mathrm {cm}}^{ - 1}$, we estimate an overall $\alpha _{\mathrm {SFG}} = 1270 {\mathrm {cm}}^{ - 1}$ at 0° polarization (Fig. 3c) and $\alpha _{\mathrm {SFG}} = 500 {\mathrm {cm}}^{ - 1}$ at 45° polarization (Fig. 3d) by fitting the results to Eq. (1). The highest SFG efficiency is achieved at a time-averaged 1.8 mW input power with 9.1 pW SFG, giving $\eta _{\mathrm {SF,wg}} = 42\% {\mathrm {W}}^{ - 1} {\mathrm {cm}}^{ - 2}$, more than three times the SHG value.

**Fig. 4:**
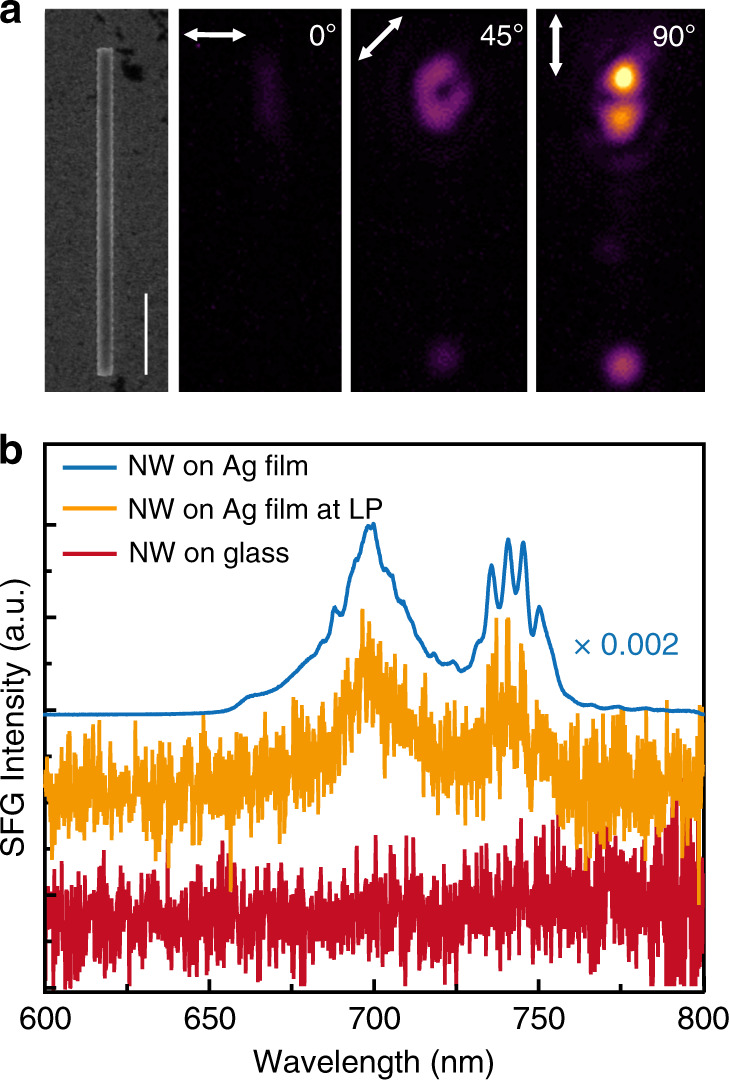
Broadband SFG on a thinner AlGaInP waveguide (113 nm × 370 nm × 8 µm)/Al_2_O_3_(6 nm)/Ag(70 nm)/SiO_2_/Si structure. **a** SEM and SFG images of the waveguide at three different polarization directions. The scale bar indicates 2 µm. **b** Measured broadband SFG spectra obtained at 90° input polarization at the output end of the waveguide for the waveguide released on Ag with an input power of 1.8 mW (blue) and a lower input power of 0.065 mW (orange) and for the waveguide released on glass (red) with an input power of 1.8 mW. The SFG obtained from the blue curve is 750 times that from the orange curve, while the signal from the orange curve is at least two times that from the red curve, giving a SFG signal ratio exceeding 1500

The advantage of using such a configuration (semiconductor waveguide-ultrathin oxide-metal film) to generate SHG and SFG signals becomes more obvious with progressively narrower waveguides, such as 113 nm × 370 nm × 8 µm (Fig. 4) and 113 nm × 250 nm × 8 µm (Fig. S7a). In contrast to the waveguides released on metal, the semiconductor waveguides of similar dimensions released on glass show undetectable SHG and SFG with a hybrid plasmonic-to-photonic signal ratio exceeding 1500 (Fig. 4b). As illustrated in Fig. S8, the edge coupling method effectively excites the TM_0_ mode at the FW, which is well confined at the semiconductor-insulator-metal interfaces even when the physical cross-section of the waveguide is on a subwavelength scale (*λ*^2^/10.8 for Fig. 4 at 1340 nm). This is in sharp contrast to the semiconductor waveguide on glass, in which optical modes cannot be effectively supported due to the diffraction limit, and the electric energy within the semiconductor on glass at the FW is only 4.1% of that within the semiconductor near metal (see section 9 of the Supporting Information). It is also found that the SFG patterns, which are observable in Fig. 3 along the plasmonic waveguide in the far field, are no longer detectable. This is caused by the decreased input coupling efficiency of the higher-order TM_1_ mode at the FW as the width of the waveguide decreases. For the hybrid plasmonic waveguides of these two widths, only the TM_0_ mode is effectively coupled into the waveguide. This is also reflected in the output spectra of Fig. S7a, where the same SFG spectra are observed at 45°, 67.5°, and 90° input polarizations, suggesting that the main contribution of the SFG is from the TM_0_ mode at the FW.

### SHG and broadband SFG on 1 μm disks

**Fig. 5:**
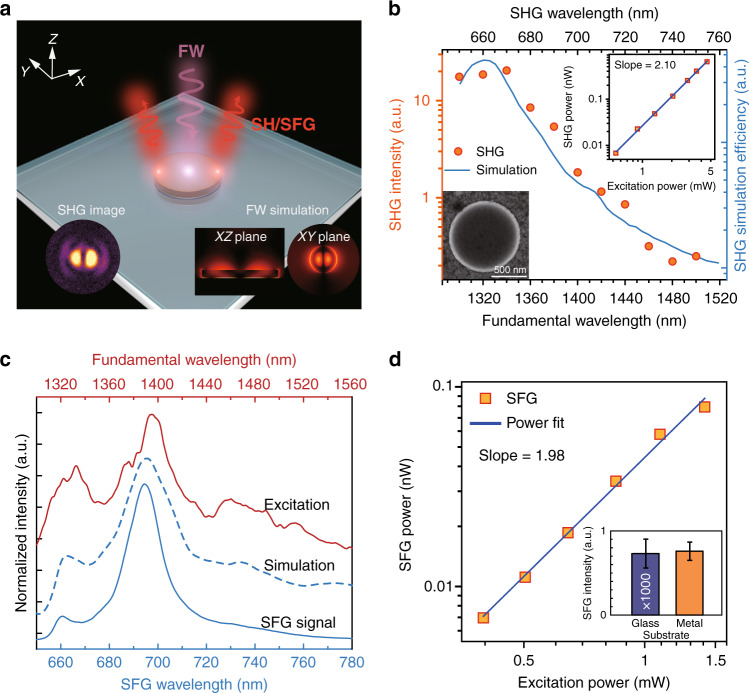
Characterization of SHG and SFG on 1 µm AlGaInP disk/Al_2_O_3_(6 nm)/Ag(70 nm)/SiO_2_/Si. **a** Diagram of the experimental setup. The insets show the experimental and simulation SHG results at 1320 nm. **b** SHG efficiency at various fundamental wavelengths with fixed input power provided by the OPO and COMSOL simulation of SHG efficiency *η* at various input wavelengths. One inset shows a log–log plot of the measured SHG power as a function of the power (1320 nm) input by the OPO and its power law fit. The other inset shows a SEM image of a typical 1 µm diameter AlGaInP disk/Al_2_O_3_(6 nm)/Ag(70 nm)/SiO_2_/Si sample. **c** Measured FW/SFG and simulated SFG spectra. **d** Log–log plot of measured time-averaged broadband SFG power as a function of power input by the SC laser and its power law fit. A comparison of the output SFG power obtained from the 1 µm disk on metal and that on glass is also given as an inset

To further demonstrate the advantage of the hybrid systems, we reduce the semiconductor waveguides to 1 µm disks. As shown in Fig. 5, wavelength-dependent SHG and broadband SFG are tested in this system.

Figure 5a depicts the experimental setup used to obtain the SHG/SFG signal on a 1 µm diameter disk released on Al_2_O_3_/Ag. Under the excitation of linearly polarized fundamental light, the SHG image appears as a two-lobe pattern (Fig. 5a inset), closely reflecting the mode distribution of the localized hybrid plasmonic mode excited at the FW, and rotates with the polarization direction (Fig. S9). As the input light is focused on the disk of subwavelength diameter, the free-space photons scatter around the perimeter of the disk. The variation in the propagation constant Δ*k* can be estimated by the Fourier transformation Δ*x*Δ*k* ∼ 1, where Δ*x* is approximated by the diameter of the disk and Δ*k* is therefore large enough to compensate for the momentum mismatch required to excite the hybrid plasmonic TM mode. The simulated electric field distribution on the XZ plane shown in Fig. 5a reveals the field confinement of the hybrid plasmonic TM mode excited at 1320 nm, similar to in the waveguided case. The inset of Fig. 5b shows a power law dependence of the SHG power on that of the FW, $P_{\mathrm {SH}} \propto P_{\mathrm {FW}}^n$, with a *n* value of 2.1 ± 0.03 at 1320 nm (FW), characteristic of the SHG process. At a given excitation power, the SHG output power sensitively depends on the FW, as shown in Fig. 5b, where the output power of SHG at 650 nm differs by a factor of 100 from that at 740 nm. Such wavelength dependence is attributed to the wavelength-dependent second-order susceptibility tensor ***d*** and the input/output response factors *f*_input_/*f*_output_. Our COMSOL simulations (see Supporting Information sections 11–12 for details) reveal a strong resonance in *f*_input_ at the FW, which we identify as the main contribution to the wavelength-dependent SHG. In the cavity case, we use

4 $$\eta _{\mathrm {SH}} = \frac{{P_{\mathrm {pk - SH}}}}{{P_{\mathrm {pk - FW}}^2}}$$

or

5 $$\eta _{\mathrm {SH}}^ \ast = \frac{{P_{\mathrm {pk - SH}}}}{{P_{\mathrm {pk - FW}}}}$$

to indicate the SHG efficiencies, as commonly used in the literature. The simulated $\eta _{\mathrm {SH}}$ is given in Fig. 5b, yielding good agreement with the experimental results.

The highest SHG intensity on the disk using an optical parametric oscillator (OPO, see “Methods”) is obtained with a time-averaged 4.6 mW input power, with a time-averaged SHG of 0.64 nW in the far field, giving $\eta _{\mathrm {SH}}^ \ast = 1.4 \times 10^{ - 7}$ at the FW of 1320 nm. Meanwhile, when a high peak power laser (optical parametric amplifier, Light Conversion SHBC-TOPAS-400-WL) is used, a SHG conversion efficiency as high as $\eta _{\mathrm {SH}} = 2.8\% {\mathrm{MW}}^{ - 1}$ or $\eta _{\mathrm {SH}}^ \ast = 2.6 \times 10^{ - 6}$ is achieved at a peak intensity of 4.8 GW cm^−2^ at 1320 nm. To the best of our knowledge, this is one of the highest SHG values reported in the literature with an FW around 1300 nm. It is worth noting that the output SHG/SFG signal is collected by using an objective of NA (numerical aperture) = 0.55. The COMSOL simulation shows a 5-fold enhancement in the output SHG power if calculated over the entire surface of the disk instead of over a collection angle in the far field (Fig. S16b).

Interestingly, after the broadband IR source is applied, the SFG conversion efficiency $\eta _{\mathrm {SF}}$, defined as:

6 $$\eta _{\mathrm {SF}} = \frac{{P_{\mathrm {pk - SF}}}}{{P_{\mathrm {pk - FW}}^2}}$$

shown in Fig. 5c, d reaches $14.8\% {\mathrm{MW}}^{ - 1}$, which is five times that of SHG. The broadband nature of the excitation light allows each FW to contribute to multiple SFG processes. Once coherently summed up, the overall SFG efficiency $\eta _{\mathrm {SF,total}}$ is greatly enhanced compared to SHG (see Supporting Information section 12). The simulated spectrum shown in Fig. 5c takes into account the wavelength-dependent input/output response factors *f*_input_/*f*_output_ and coherent generation and summation of the SFG signal over a wavelength span of 40 nm for the FW, reproducing the shape of the experimental results. The total simulated time-averaged power over the wide wavelengths (1300 nm to 1600 nm) is summed to be a time averaged 0.065 nW with an input power of 1.2 mW, agreeing very well with the measured results. The excitation of the strongly localized hybrid plasmonic TM mode induces an *E*_z_ component in the semiconductor region that is perpendicular to the metal surface. The metal responds to the oscillation of the *E*_z_ field with image charges and produces an image dipole that aligns in the same direction as the dipole moment in the semiconductor. As a result, the electric field in the semiconductor near the metal is strongly enhanced. In the photonic cavity, neither an out-of-plane component nor enhancement exists. The total electric field at the FW within the semiconductor material near the metal is approximately ten times the value within the structure released on glass (see Supporting Information section 13). Consequently, the SFG signal obtained from the 1 µm disk near Ag exhibits a 1039 times enhancement compared to that released on glass at a time-averaged input power of 1.8 mW (inset of Fig. 5d and Fig. S13c).

## Discussion

For both waveguide and cavity configurations, a high SHG/SFG efficiency is achieved due to the effective excitation of hybrid plasmonic TM modes at the FWs. Compared to pure photonic structures, the hybrid plasmonic modes can be confined into a much thinner geometry, with a nonlinear material thickness of ∼*λ*/12, and the metal response strongly enhances the out-of-plane component of the *E* field in the semiconductor region. For the waveguide with a cross-section of 113 nm × 250 nm (thickness × width), its physical cross-sectional area of *λ*^2^/16 has a TM_0_ mode area *A*, defined as:

7 $$A = \frac{{{\int\!\!\!\!\!\int} {W({\boldsymbol{r}})d^2{\boldsymbol{r}}} }}{{Max\left( {W\left( {\boldsymbol{r}} \right)} \right)}}$$

on the deep subwavelength scale of *λ*^2^/135 (*W*(***r***) is the electric energy density of the mode) at the FW of ~1340 nm. This extremely confined fundamental mode allows for effective SHG and SFG in this ultrathin plasmonic waveguide (113 nm × 250 nm × 8 µm). A SFG conversion efficiency of 14.0% ${\mathrm {W}}^{ - 1}{\mathrm {cm}}^{ - 2}$ at 90° input polarization is derived at a time-averaged input power of 1.8 mW, while pure photonic waveguides of the same dimensions completely lose mode confinement and produce no detectable signal. The 1 μm disk results further demonstrate the advantage of the hybrid configuration, as efficient SHG/SFG is achieved with a mode volume *V*, defined as:

8 $$V = \frac{{{\int\!\!\!\!\!\int\!\!\!\!\!\int} {W({\boldsymbol{r}})d^3{\boldsymbol{r}}} }}{{Max\left( {W\left( {\boldsymbol{r}} \right)} \right)}}$$

as small as *λ*^3^/206 at the FW of 1320 nm.

In conclusion, we have studied the SHG and broadband SFG in top-down lithographically defined AlGaInP nano(micro)structures in the vicinity of Ag and compared them with those in structures of the same dimensions released on glass. Our results suggest that the hybrid plasmonic structures possess great advantages over their photonic counterparts when any dimension of the structure is on the deep subwavelength scale. The excitation of hybrid plasmonic modes at the FW tightly confines the light into a small region near the semiconductor-insulator-metal interface and allows for highly effective SHG and SFG. This advantage is clearly demonstrated in the waveguide configuration with a mode area of λ^2^/135 and in the resonant cavity case with a mode volume of λ^3^/206, whereas the photonic waveguides and cavities can only produce barely detectable or undetectable SHG and SFG due to the lack of mode confinement. The underlying origin of the high efficiency in waveguides is demonstrated by the direct visualization of SHG/SFG phase matching by virtue of leaky modes. We also show that the conversion efficiency of the broadband SFG in the disks can be substantially increased compared to narrowband SHG by exploiting the multiple SFG processes offered by a coherent broadband light source. We believe that our study, based on precisely tailored nanostructures, further enriches the application toolbox of plasmonic cavities and waveguides in integrated plasmonic circuits and will promote the development of on-chip hybrid plasmonic nanodevices for quantitative nonlinear optics applications.

## Materials and methods

### Sample fabrication and optical characterization

AlGaInP nanostructures (disks and waveguides with rectangular cross-sections) were fabricated and released onto Al_2_O_3_ (6 nm)/Ag (70 nm)/SiO_2_/Si and glass substrates using the methods detailed in ref. ^33^. As discussed in refs. ^25,26^, III–V compounds fabricated in this way exhibit strong second-order optical nonlinearity.

Super continuum (SC) light (NKT SC-400, 40 MHz, pulse width 76 ∼ 90 ps, 450 nm to 2.5 µm) or single wavelength tunable light output from an optical parametric oscillator (OPO, A. P. E Levante Emerald 690 nm to 2 µm) pumped by a frequency doubled optical fiber laser (Spark Antares, 80 MHz, pulse width 4 ∼ 5 ps, 1064 nm and 532 nm) was focused onto the sample by a long working distance ×50 VIS-IR objective (Olympus MIRPLAN50, 0.55 NA), as shown in Fig. S1. For the broadband SC input, multiple filters (Thorlabs FEL0900 and DMLP1180 as F1 in Fig. S1) were used before the IR light entered the microscope. The beam splitter cube in the microscope is a BS016 from Thorlabs. The reflected light is collected by the same objective and passes through an 800 nm short-pass filter (Thorlabs FES0800 as F2 in Fig. S1) before being directed to either a charge-coupled device (CCD) (AVT Prosilica GC1290) for imaging or a spectrograph (Andor Kymera193i) equipped with a visible CCD (Andor Newton DU920P) and an IR sensor array (Andor InGaAs DU490A-1.7). To obtain the maximum SHG power from the 1 µm AlGaInP disk released on the Al_2_O_3_ (6 nm)/Ag (70 nm)/SiO_2_/Si substrate, an optical parametric amplifier system (OPA, Light Conversion SHBCTOPAS-400-WL) delivering a 4 picosecond signal tunable between 400 nm and 4 μm pumped by a femtosecond regenerative Ti-sapphire amplifier (Coherent LIBRA, 1 kHz, 800 nm) was used to excite the structures, and a photomultiplier tube (Hamamatsu E717-500) with a lock-in amplifier was used to obtain the SHG signal.

## Supplementary information

Supporting Information

## Data Availability

The data and information within this paper are available from the corresponding author upon request.
