# Supplementary material for: Direct visualization of phase-matched efficient second harmonic and broadband sum frequency generation in hybrid plasmonic nanostructures
Source: Light Sci Appl. 2020 Oct 22;9:180. doi: 10.1038/s41377-020-00414-4 (PMC7582155; doi:10.1038/s41377-020-00414-4)
Supplement: Supplementary file 1 — Supporting Information [file 41377_2020_414_MOESM1_ESM.pdf]

Supporting Information for

Direct visualization of phase-matched efficient second harmonic and broadband sum frequency generation in hybrid plasmonic nanostructures

Zhe Li[1,2], Brian Corbett[3], Agnieszka Gocalinska[3], Emanuele Pelucchi[3], Wen Chen[4], Kevin. M. Ryan[5], Pritam Khan[1], Christophe Silien[1], Hongxing Xu[2], Ning Liu[1*]

[1] *Department of Physics and Bernal Institute, University of Limerick, Ireland*

[2] *The School of Physics and Technology, and Institute for Advanced Studies and Center for Nanoscience and Nanotechnology, Wuhan University, Wuhan, 430072, China*

[3] *Tyndall National Institute, University College Cork, Ireland*

[4] *Institute of Physics, École Polytechnique Fédérale de Lausanne (EPFL), CH-1015 Lausanne, Switzerland*

[5] *Department of Chemical Sciences and Bernal Institute, University of Limerick, Ireland*

1. Experimental setup and choices of input fundamental wavelengths

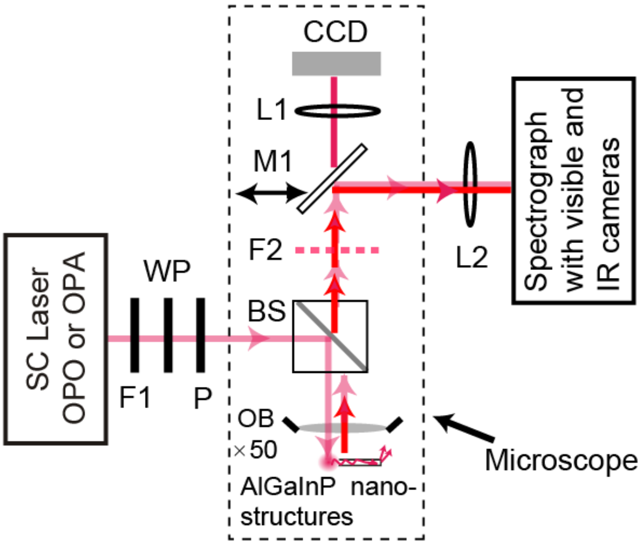

Fig. S1. Diagram of the optical setup. BS = beam splitter, M = mirror, F = filter, L = lens, WP = wave plate, OB = objective and P = polarizer. The light red indicates the input IR beam and red indicates the output SHG/SFG signal.

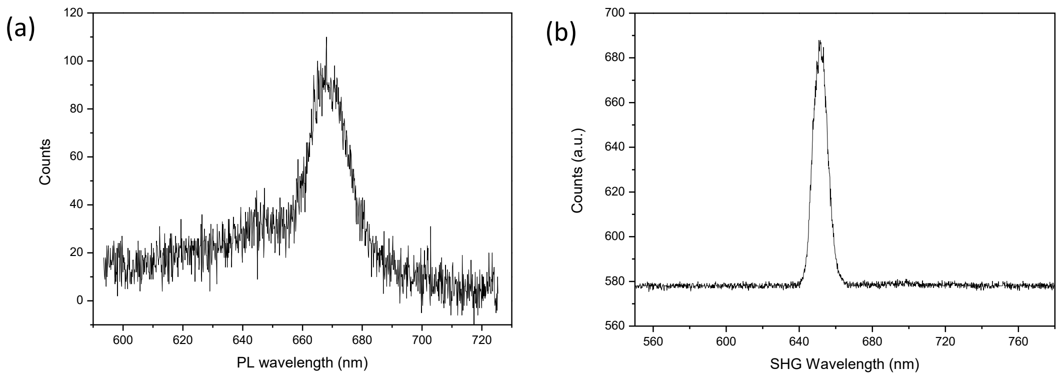

Fig. S2. (a) A typical PL of the AlGaInP material when excited at 570 nm. Panel (b) shows that when the sample is excited at 1300 nm, the SHG is centred around 650 nm and does not cause re-emission of PL. For any wavelength longer than 1300 nm, we believe that they only cause SHG or SFG processes in the materials and the re-absorption of SHG and SFG at certain wavelengths.

2. Comsol simulation

Wave Optics module in COMSOL MULTIPHYSICS 5.2a simulation package was used to simulate the SHG in frequency domain. To accurately model the SHG intensity collected by the objective in the far field, a 3D model was used. A 3D Gaussian beam (beam waist w_0 is set the same value as λ/2NA, λ the fundamental wavelength) was applied as background field to approximate the excitation beam. The SHG process is entered by the nonlinear polarization vector (discussed in details in section 11) and the frequency domain stationary solutions were obtained for a series of simulation parameters (see Fig. S8 for the examples of E field distribution at the FW and Fig. S4 for the examples of E field distribution at the SHG frequency). In order to reduce the calculation time and yet still capture the essential physics, the simulations were run with scattering boundary condition and without perfect matching layers. For the calculations of resonant modes in a cavity at the FW, the eigenfrequency solver was used.

3. Beat patterns observed on 113 nm × 580 nm × 8 μm AlGaInP waveguide released on Al_2O_3/Ag at 45° input polarization

As mentioned in the main texts, the period of the beat pattern observed on 113 nm × 584 nm × 8 μm AlGaInP waveguide released on Al_2O_3/Ag at 45° input polarization is not sensitive to the wavelength from 1300 nm to 1500 nm. This effect comes from the dispersion relationship of TM_0 and TM_1 mode at the fundamental wavelengths, as shown in the figure below. The periodicity of the beam pattern generated by the combination of TM_0 and TM_1 modes can be approximated by $2\pi/(k_{TM0}\text{-}k_{TM1}) = \lambda_0/(n_{TM0}\text{-}n_{TM1})$. From 1300 nm to 1500 nm, the periodicity varies from 2.35 μm to 2.45 μm, corresponding to only a 4.3% change. This is the reason we called them 'insensitive'. The effective refractive indices in Fig. S3 is obtain by COMSOL eigenfrequency solver.

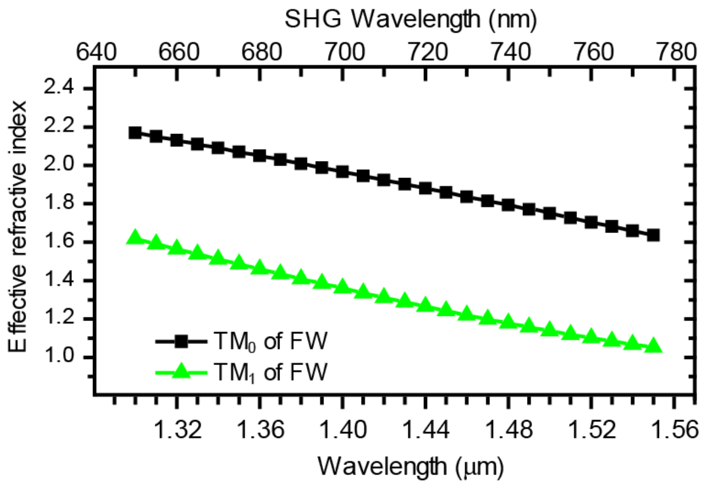

Fig. S3 COMSOL simulation on effective refractive index of TM_0 and TM_1 mode excited in the 113 nm × 584 nm × 8 μm AlGaInP waveguide released on Al_2O_3/Ag at 45° input polarization.

4. Optical intensity evolution of FW and SHG along propagation distance simulation

The optical intensity evolution is simulated following reference [1,2], where FW and SH are calculated by solving the coupled-mode equations:

$$\frac{dA_{FW}}{dz} = -\alpha_{FW}A_{FW} + i\frac{\omega_{FW}}{4}\varkappa_1 A_{FW}^* A_{SH}\exp\,(i\Delta\beta z) \qquad \text{(S1)}$$

$$\frac{dA_{SH}}{dz} = -\alpha_{SH}A_{SH} + i\frac{\omega_{FW}}{4}\varkappa_2^* A_{FW}A_{FW}\exp\,(-i\Delta\beta z) \qquad \text{(S2)}$$

Here A_{FW} and A_{SH} are the mode amplitudes at FW and SH, respectively. α_{FW} and α_{SH} are the electric field loss coefficients at FW and SH, respectively. Z is the propagation direction and $\Delta\beta$ the phase mismatch constant. The nonlinear coupling efficiency (NCC) $\varkappa_{1,2}$ is calculated using the following equations[1]:

$$\varkappa_1 = \varepsilon_0 \iint \left[\mathcal{X}^{(2)} : E_{SH}(x,y)E_{FW}^*(x,y)\cdot E_{FW}'(x,y)\right]dxdy$$

$$\varkappa_2 = \varepsilon_0 \iint \left[\mathcal{X}^{(2)} : E_{FW}(x,y)E_{FW}(x,y)\cdot E_{SH}'(x,y)\right]dxdy$$

where E_{FW} and E_{SH} are the normalized electric field of modes at FW and SH, respectively.

Assuming $|A_{SH}| << |A_{FW}|$, which is the case in current experimental condition, the 2nd term on the right side of Eq. (S1) is ignored. If we further assume the phase matching condition is satisfied, the solution to Eq. (S1) and (S2) can then be obtained analytically[2]:

$$A_{FW}(z) = A_{FW}(0)\exp(-\alpha_{FW}z),$$

$$A_{SH}(z) = \frac{i\omega_{FW}\varkappa_2^* {A_{FW}}^2(0)}{4(\alpha_{SH} - 2\alpha_{FW})}\left[\exp(-2\alpha_{FW}z) - \exp\,(-\alpha_{SH}z)\right]$$

The change of SHG amplitude along z direction is then essentially determined by the two loss constants. The A_{SH} maximum occurs at location where $\frac{dA_{SH}}{dz} = 0$, with expression given in Eq. (1) of the main texts. Fig. S4(a) gives the A_{SH} maximum as a function of α_{SH}, at two different α_{FW} values. Fig. S4(b) shows the SH electric field $|E_{SH}|$ distribution at 670 nm,

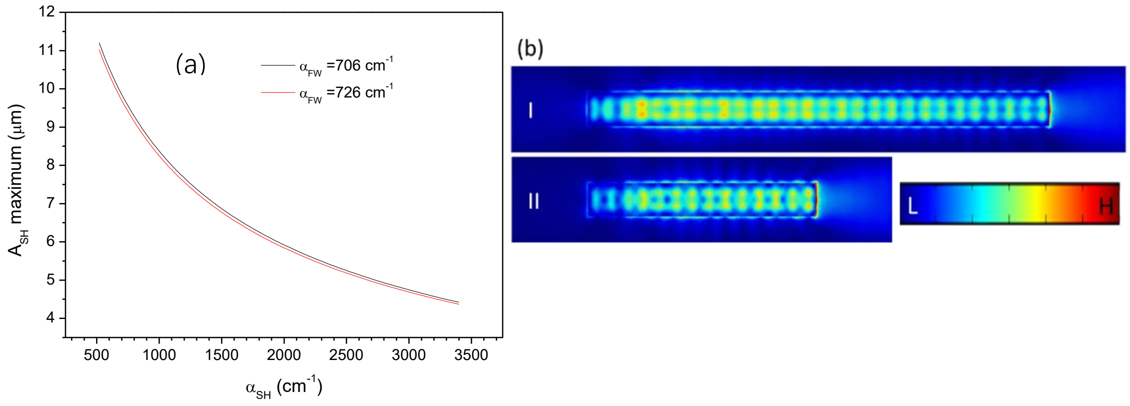

where the input FW is polarized along the long axis of the waveguide (90°) and edge coupled into the waveguide from the left side, with (I) of 8 μm in length and (II) 4 μm in length.

Fig. S4 (a) A_{SH} maximum as a function of α_{SH}, at $\alpha_{FW} = 706\, cm^{-1}$ and $\alpha_{FW} = 726\, cm^{-1}$. (b) SH electric field $|E_{SH}|$ distributions at 670 nm at the top surface of the waveguide. The input FW is polarized along the long axis of the waveguide and edge coupled into the waveguide from the left side, with (I) of 8 μm in length and (II) 4 μm in length and a cross section of 113 nm × 584 nm.

5. SFG and SHG conversion rates

Calibration of the collection system

In all experiments, we used the same CCD (AVT Prosilica GC1290) to take images. By comparing the quantum efficiency data provided by the company with the SFG spectrum (Fig. 5(c)), we understand that the SFG spectrum is mostly centred around 680 nm to 710 nm, where the quantum yield of the CCD is between 34% to 29%.

To calibrate the SFG output power, we used a band pass filter (740 nm ± 10 nm) to filter out a narrow band input light at 740 nm (quantum yield 23.9) from the supercontinuum source. We then used the power meter to measure the laser powers before entering the microscope and after the objective (on the sample) to establish the ratio of light being delivered to the sample. We know from other experiments that the reflectivity of the sample is around 0.9. We then focused the laser around 740 nm onto our sample (on the smooth region) and the focused light spot is recorded by the CCD camera. By integrating the output reading over the region of light spot on the CCD image, we can further establish the relationship between the power of light being delivered to the sample and the total integrated reading from the CCD. We use this relationship to approximate the power of collected SFG signal from the CCD image. This calibration method, however, overestimates the collected SFG power by (34+29)/23.9/2-1 = 31.8%, which is considered in the final conversion efficiency.

The SHG experiments were carried out by the OPO system. In this case, we use the 660 nm red laser pointer. Similar relationship of input laser power at 660 nm with the integrated light spot on the CCD image was obtained. In SHG cases, the calibrated SHG power is normalized against the quantum efficiency difference at various wavelengths of the CCD camera. One point that needs to be noted is that as shown in Fig. S16(b), the objective only collects about 1/5 of the generated SHG. In both SFG and SHG cases, the calibrated results only account for the 'collected' signal, not the really emitted total signal.

Conversion efficiency of disks

The commonly used SHG conversion efficiency η_{SH}^* for a cavity is defined as $P_{pk\text{-}SF}/P_{pk\text{-}FW}$ or $\eta_{SH} = \frac{P_{pk-SF}}{P_{pk-FW}^2}$. For the 1 µm disk, using the OPO (80 MHz, 5 ps pulse duration), we measured a time-averaged 4.6 mW generating 0.64 nW SHG, with $\eta_{SH} = 1.2 \times 10^{-8}\, W^{-1} = 1.2\%\ \mathrm{MW}^{-1}$ or $\eta_{SH}^* = 1.4 \times 10^{-7}$ at peak input intensity of 760 MW cm^{-2}. Using the OPA (1 KHz, 4 ps pulse duration), we measured a time-averaged 0.38 µW generating 1 pW SHG, with $\eta_{SH}^* = \eta_{SH} = 2.8 \times 10^{-8}\, W^{-1} = 2.8\%\ \mathrm{MW}^{-1}$ or 2.6×10^{-6} at peak input intensity of 4.8 GW cm^{-2}. The Super Continuum (SC) laser has a repetition rate of 40 MHz, with a pulse duration around 76 to 90 ps. After calibration, we have a time-averaged 1.19 mW (peak input power 250 mW) generating 0.063 nW SFG, with $\eta_{SFG} = 14.8 \times 10^{-8}\, W^{-1} = 14.8\%\ \mathrm{MW}^{-1}$ or $\eta_{SFG}^* = 5.3 \times 10^{-8}$ at the peak input intensity of 15 MW cm^{-2}.

Conversion efficiency of waveguides

In the waveguide case, the conversion efficiency is defined as $\eta_{SH,wg} = \frac{P_{pk-SH,wg}}{P_{pk-FW,wg}^2 L^2}$. Here $P_{pk-FW,wg}$ is the waveguided input peak power, $P_{pk-SH,wg}$ the waveguided output SHG peak power and *L* the length of the waveguide. To measure the coupling efficiency of a waveguide, we first measured the reflected power of input light on a smooth region of the substrate, which we call value *a*. We then measured the reflected power of input light when the light spot was focused on the middle of the waveguide, which we call value *b*. The value (*a*-*b*) approximates the scattered light due to the presence of the waveguide. In the 3rd step, we positioned the input light spot at the coupling end of the waveguide and measure its reflected power, which we call value *c*. We approximate the input light that was coupled into the waveguide by (*a*-*c*)-(*a*-*b*)/2 =*a*/2+*b*/2-*c* and the coupling efficiency would be ((*a*+*b*)/2-*c*)/*a*. This method, however, still overestimates the input light that couples into the waveguides, meaning that we counted more input light than that was really coupled into the waveguide, but this is the closest value we can obtain from the experiments. The measured input coupling efficiency varies with the input polarization for the 113 nm × 584 nm × 8 µm waveguide, with 18.6% for TM_0 FW mode, 9.3% for FW TM_1 mode and 12.1% for 45° polarization. For input fundamental wave polarized along the long axis of the waveguide of 113 nm × 584 nm × 8 µm, we obtained time-averaged 0.36 nW waveguided SHG signal in the far field from 7.6 mW input power at 1340 nm using OPO, giving a $\eta_{SH,wg} = 12\%\ W^{-1}cm^{-2}$. For a shorter waveguide of 113 nm × 584 nm × 4 µm, the expected $\eta_{SH,wg}$ can be further increased to $96\%\ W^{-1}cm^{-2}$. For the broad band SFG, we measured SFG signal of 9.1 pW at 1.8 mW input power using SC laser, giving a $\eta_{SF,wg}$ =42% $W^{-1}cm^{-2}$. For the 113 nm × 250 nm × 8 µm waveguide, the

input coupling efficiency is only 8.3% for TM_0 FW mode, with 0.60 pW at 1.8 mW input power, with the $\eta_{SF,wg}$ =14% $W^{-1}cm^{-2}$.

6. Diagram of SHG and SFG

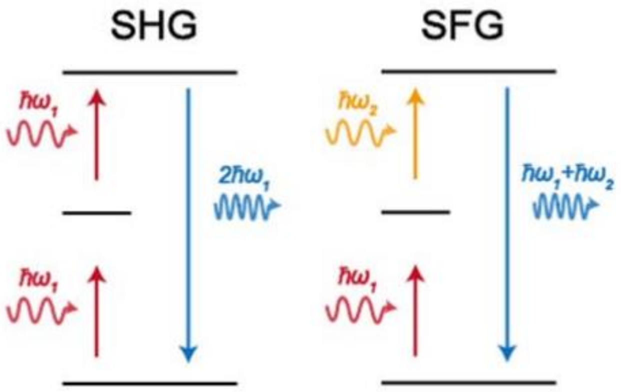

Fig. S5 Diagram demonstrating of SHG, with two photons of the same frequency, and SFG processes with two photons of different frequencies.

7. SFG spectrum at the end of 113 nm × 584 nm × 8 µm waveguide

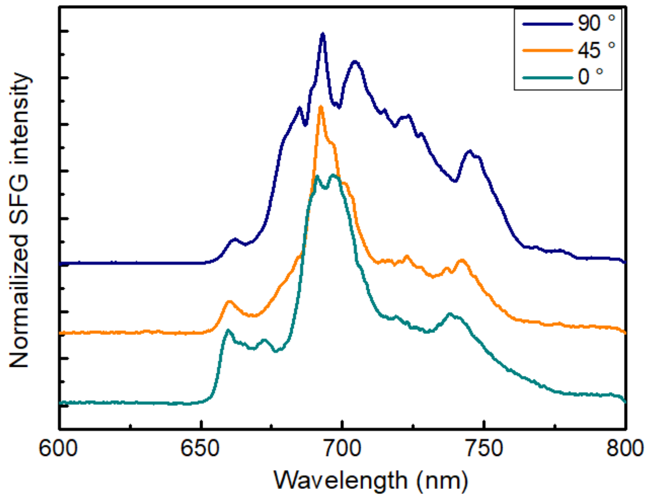

Fig. S6 SFG spectrum of 113 nm × 584 nm × 8 µm waveguide at different excitation polarization.

8. SFG on thinner (in width) waveguides released on Al_2O_3/Ag

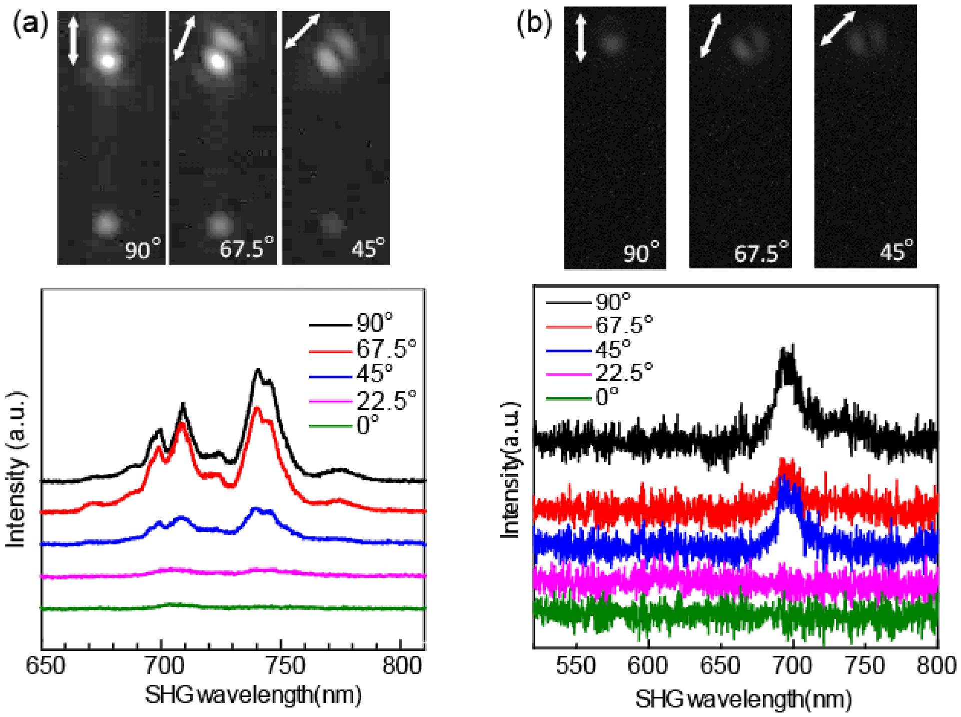

Fig. S7 SFG images and spectra obtained at output end at various wavelength for waveguide with dimensions (thickness × width × length) of 113 nm × 250 nm × 8 μm (a) and 113 nm × 190 nm × 8 μm.

Once the width of the waveguide is further decreased down to 190 nm (Fig. S7(b)), waveguided SFG signal can only be achieved at the polarization > 45°.

9. Comparison of FW excitation in plasmonic and photonic waveguides by edge coupling

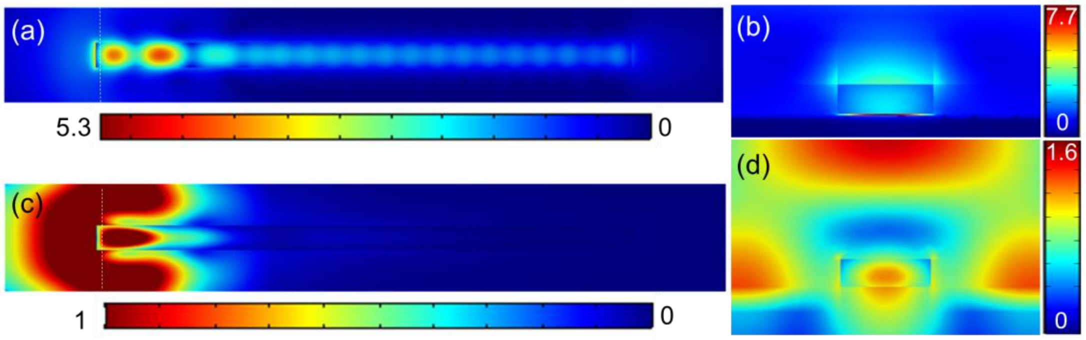

Fig. S8 COMSOL simulation on the field distribution at the FW of 1340 nm in the hybrid plasmonic and photonic waveguides of 113 nm × 370 nm × 8 μm upon the edge coupling. In both cases, the input Gaussian beam is focused at the left edge of the waveguide. (a, c) The |E| field distribution on a plane half way between the top surface of the waveguide and the substrate and parallel to the substrate for the hybrid plasmonic (released on Al_2O_3/Ag) and photonic (released on glass) waveguides respectively. (b, d) |E| field distribution at the cross section of the waveguide at locations defined by the dashed white lines in (a) and (c) respectively. It is

clear that the hybrid plasmonic mode excited in the waveguided in the metal is well confined and supported by the waveguide. In the photonic case, no waveguided mode can be identified.

10. Polarization dependence SHG image

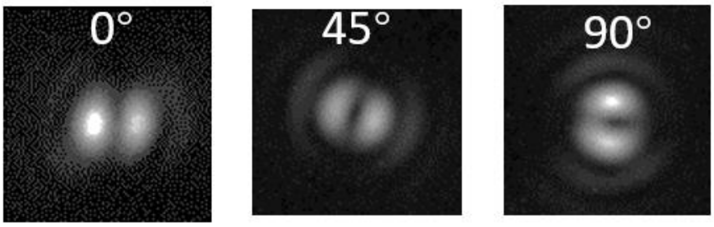

Fig. S9 Polarization dependence SHG image at FW of 1320 nm.

11. Second order susceptibilities tensor for input wavelengths from 1300 nm to 1600 nm

The second order susceptibilities tensor ***d*** are obtained from ref [3]. As detailed in ref. [3], the naturally cleaved facets of ALGaInP thin film grown on AlGaAs/GaAs are (110) and ($\bar{1}$10) facets. The fabricated waveguides are therefore aligned either along [110] or [$\bar{1}$10] direction, which is later determined by the polarization dependant second harmonic generation (SHG) intensity study on the circular AlGaInP/GaInP/AlGaInP disks released on Al_2O_3/Ag. According to ref. [3], we identify our lab frame second order susceptibilities tensor as:

$$\boldsymbol{d}=\begin{bmatrix} 0 & 0 & 0 & 0 & d'_{15}+d'_{14} & 0 \\ 0 & 0 & 0 & d'_{15}-d'_{14} & 0 & 0 \\ d'_{31}+d'_{14} & d'_{31}-d'_{14} & d'_{33} & 0 & 0 & 0 \end{bmatrix} \text{ or}$$

$$\boldsymbol{d}=\begin{bmatrix} 0 & 0 & 0 & 0 & d'_{15}-d'_{14} & 0 \\ 0 & 0 & 0 & d'_{15}+d'_{14} & 0 & 0 \\ d'_{31}-d'_{14} & d'_{31}+d'_{14} & d'_{33} & 0 & 0 & 0 \end{bmatrix},$$

depending on the long axis of the waveguide along [110] or [$\bar{1}$10] direction. The value of d'_{14} is chosen as 110 pm V^{-1}, d'_{31} 27.5 pm V^{-1}, d'_{33} 60 pm V^{-1} and d'_{15} -15.2 pm V^{-1} at 1500 nm [3]. $\varepsilon_0\boldsymbol{d}$ gives the value used in simulation with the unit of C/V^2. The nonlinear polarization vector ***P*** is then written as:

$$\begin{bmatrix} P_{2,x} \\ P_{2,y} \\ P_{2,z} \end{bmatrix}=\varepsilon_0\begin{bmatrix} 0 & 0 & 0 & 0 & d'_{15}+d'_{14} & 0 \\ 0 & 0 & 0 & d'_{15}-d'_{14} & 0 & 0 \\ d'_{31}+d'_{14} & d'_{31}-d'_{14} & d'_{33} & 0 & 0 & 0 \end{bmatrix}\begin{bmatrix} E_{1,x}^2 \\ E_{1,y}^2 \\ E_{1,z}^2 \\ 2E_{1,y}E_{1,z} \\ 2E_{1,x}E_{1,z} \\ 2E_{1,x}E_{1,y} \end{bmatrix} \quad \text{(S3)}$$

This vector is entered in the COMSOL for SHG simulations as discussed in section 2. To obtain an analytical expression for the polar pattern, all three components of electric field at the FW need to be considered. In current experimental setup, the input beam is focused on the sample with mostly x and y components, where we can define $E_{1x}= E_0\cos(\theta-\theta_0)$ and $E_{1y}= E_0\sin(\theta-\theta_0)$. Due to the excitation of the hybrid plasmonic mode at the FW, z component cannot

be ignored in the calculation, where we define $E_{1z} = \alpha E_0$ and α is the input geometric factor. Once these three components are plugged into Eq. (S3), the nonlinear polarization vector $\boldsymbol{P}$ can be obtained and also has three components. The total SHG emission power P_{SH} is determined by $P_{2,x}^2 + P_{2,y}^2 + \beta P_{2,z}^2$, where β is the output geometric factor. Base on above analyses,

$$P_{SF} \propto 4\alpha^2(d_{15}'^2 + d_{14}'^2) + \beta(d_{31}' + \alpha^2 d_{33}')^2 + \beta d_{14}'^2 \cos\big(2(\theta - \theta_0)\big)^2 + (2\beta(d_{31}' + \alpha^2 d_{33}') + 8\alpha^2 d_{15}')d_{14}' cos(2(\theta - \theta_0)).$$

We can define $A = \frac{\beta d_{14}'^2}{4\alpha^2(d_{15}'^2 + d_{14}'^2) + \beta(d_{31}' + \alpha^2 d_{33}')^2}$, $B = \frac{(2\beta(d_{31}' + \alpha^2 d_{33}') + 8\alpha^2 d_{15}')d_{14}'}{4\alpha^2(d_{15}'^2 + d_{14}'^2) + \beta(d_{31}' + \alpha^2 d_{33}')^2}$ and simplify the SHG emission power P_{SH} as $P_{SF} \propto 1 + A\cos\big(2(\theta - \theta_0)\big)^2 + Bcos(2(\theta - \theta_0))$.

At fundamental wavelengths that are not 1500 nm, we scale all d_{ij} values against those obtained at 1500 nm through a calibration sample, a thin AlGaInP structure released on glass. As shown in Fig. S10(a), the second harmonic generation (SHG) is measured through the calibration sample in transmission by a photomultiplier tube at different fundamental wavelengths while the input power is fixed. The lateral dimension of the structure was chosen to be over 5 µm × 30 µm, much larger than the beam diameter so the size effect can be ignored. The measured SHG signal difference is therefore attributed to the d_{ij} value change at various wavelengths. To remove the contribution from the interference through the thin AlGaInP layer, COMSOL simulation is run on the structure of 115 nm thick to obtain the SHG values with fixed d_{ij} values (at 1500 nm) to show the effect due to fixed thickness at various wavelengths (Fig. S10(a)). The square root of ratio of the measured SHG/simulated SHG gives the contribution from the d_{ij} values at various wavelengths, as shown in Fig. S10(b). Here, the *d* ratios are normalized against the values at 1500 nm, at which wavelength the ratio is set to 1. It is clear from the fitting curve that *d* values increase when the wavelengths coincide with the photoluminescence emission band of AlGaInP. The dependence of *d* values on wavelength is considered in all SHG and SFG simulations in the paper.

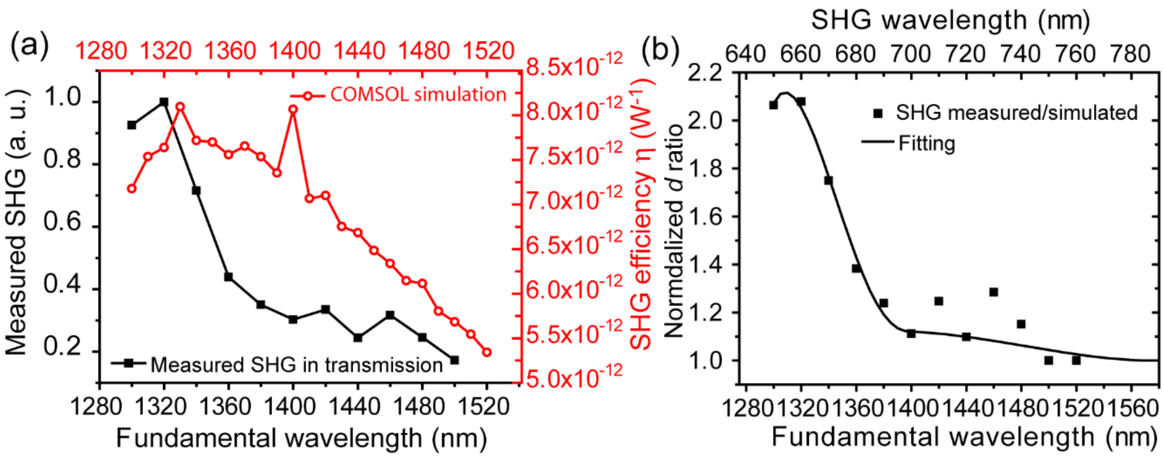

Fig. S10 (a) Measured SHG through a thin AlGaInP film (115 nm) released on glass with lateral size > 5 µm × 30 µm in transmission configuration and COMSOL simulation on obtained SHG signal using fixed ***d*** tensor elements (values from ref.[3]) at various input wavelengths. (b) Normalized

multiplication factor to ***d*** tensor elements against values obtained from reference and its fitting. The fitting curve shows a peak coincides with the photoluminescence band of AlGaInP. (Ref. [4] and Fig. S2(a))

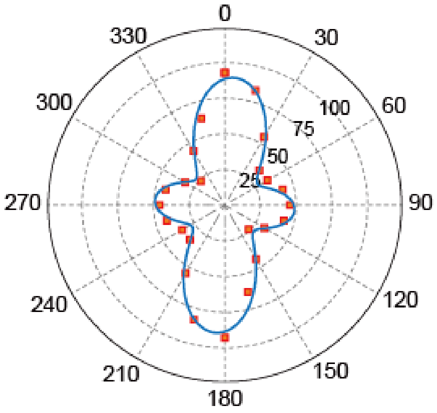

Fig. S11 Polar plot of measured SFG power as a function of fundamental input polarization.

12. Input and output responses in SHG and SFG for the disks

The overall SHG can be considered as the combination effects of input response funnction f_{input} with $f_{input} = \frac{\iiint W_{e,\ FW} dV}{P_{FW}}$ and output response function $f_{output,\ SHG}$ with $f_{output,SHG} = \frac{\iint \vec{S}_{SHG} \cdot d\vec{A}}{(\iiint W_{e,\ FW} dV)^2}$. The designed thickness of the AlGaInP structures is 110 nm. However, the last etching step before releasing the structures to Al_2O_3/Ag substrate could have slightly over etched or under etched the structures. To find out the simulation results that fits the best to the experiments, various thicknesses around 110 nm were examined by simulations. Fig. S12 shows the simulated f_{input} (Fig. S12(a)) and $f_{outpu,SHG}$ (Fig. S12(b)) respectively of a 1 µm diameter AlGaInP disk released on the Al_2O_3/Ag/SiO_2/Si substrate at various disk thickness from 90 nm to 125 nm. Using this method, we can evaluate the contributions from the fundamental input beam and that from the SHG separately. The overall conversion efficiency η_{SH} for a cavity is defined as $\eta_{SH} = \frac{P_{pk-SH}}{P_{pk-FW}^2} = f_{input}^2 f_{output}$, which is plotted in Fig. S12 (c). We can see from the plots that when the thickness increases, there is a trend showing resonant peaks emerging in output response factor f_{output}. These peaks, however, are located at different wavelengths relative to the input response. As a result, the overall conversion efficiency response become a broader band with multiple peaks and a 50% to 60% increase in overall conversion efficiency. By comparing the simulation results with our experimental data, we identify that the thickness around 105 nm gives the best fit to the experimental results. Fig. S12(d) and (e) shows the |E| field distribution of the resonant mode at 1320 nm at the thickness of 105 nm.

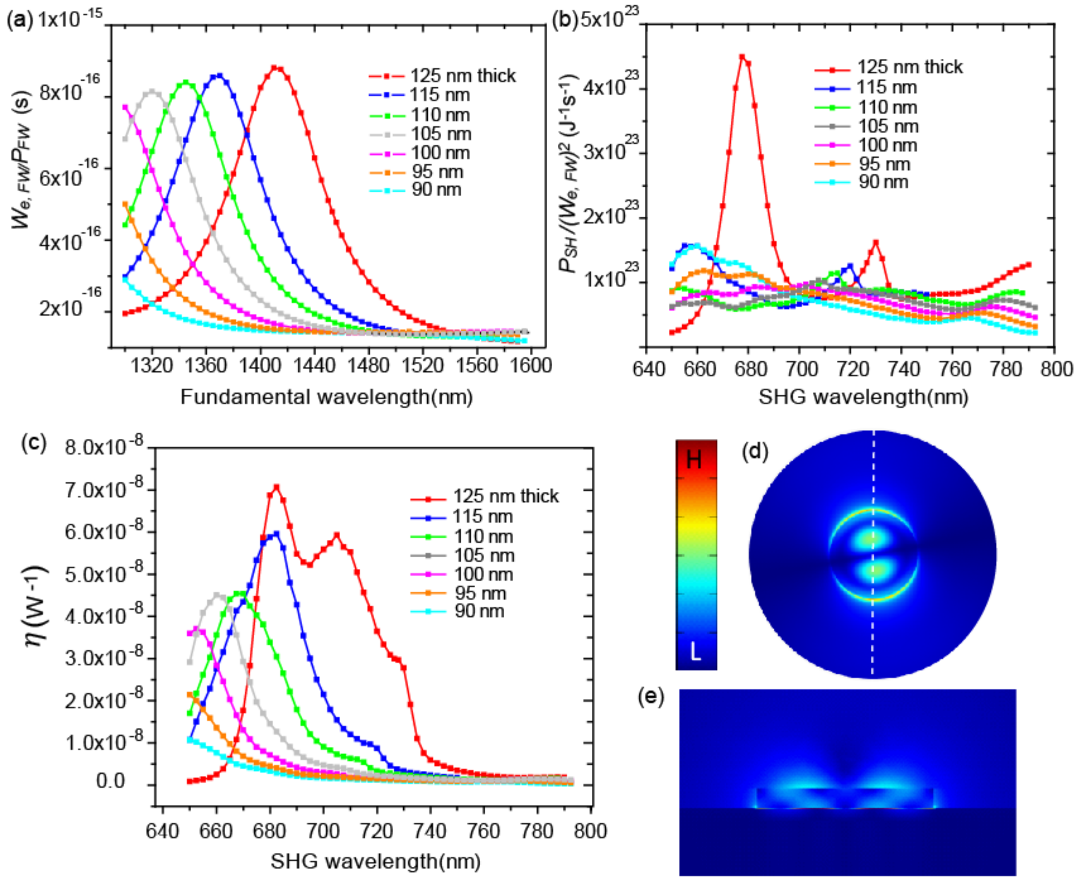

Fig. S12 COMSOL simulations on the input enhancement factor f_{input} (a), the output scattering factor f_{output} (b) and the overall conversion efficiency η_{SH} (c) at different AlGaInP thicknesses with diameter of the disk set as 1 μm. (d) The |E| distribution of the localized hybrid plasmonic resonant mode excited at the FW of 1320 nm on the top surface of the 1 μm disk and (e) that on the yz plane defined by the dashed white line in (d).

Similarly, we can develop the f_{input} and f_{output} for SFG processes as well. In this case, f_{input} is defined the same as that in SHG case, while $f_{output,\ SFG}$ is defined as $f_{output,SFG} = \frac{\iint \vec{S}_{SFG} \cdot d\vec{A}}{(\iiint W_{e,\ FW1} dV)(\iiint W_{e,\ FW2} dV)}$, which includes two fundamental wavelengths FW_1 and FW_2.

SFG process with a broadband coherent source

For a broadband light source of *n* different wavelengths, the nonlinear polarisation P_{SFG} is:

$$P_{SFG} = \varepsilon_0 d\left(\sum_{i=1}^{n} E(\omega_i)\right)^2 = \varepsilon_0 d\left(\sum_{i=1}^{n} E^2(\omega_i) + 2\sum_{i=1}^{n}\sum_{j=i+1}^{n} E(\omega_i)\, E(\omega_j)\right)$$

Here we assume the scalar form for simplicity. If assuming $E(\omega_i) = E_0 e^{-i\omega_i t}$ and the resulted SFG frequencies are different, we have the intensity of the FW and SFG as:

$$I_{FW} = \sum_{i=1}^{n} |E(\omega_i)|^2 = n\, E_0^2$$

$$|P_{SFG}|^2 = {\varepsilon_0}^2 d^2 (n + 2n(n-1)) E_0^2 = {\varepsilon_0}^2 d^2 (2n^2 - n) E_0^4$$

For the narrow band light source at a single frequency ω with an intensity $I_{FW} = nE_0^2$, the electric field $E(\omega) = \sqrt{n}E_0 e^{-i\omega t}$, the $P_{SHG} = \varepsilon_0 d E^2(\omega)$ and $|P_{SHG}|^2 = {\varepsilon_0}^2 d^2 n^2 E_0^4$

In this simple demonstration, we show that the $\frac{|P_{SFG}|^2}{|P_{SHG}|^2} \rightarrow 2$ at the same input fundamental power.

To obtain ratio larger than 2, some special condition is required for the input light source. This is shown in the equation $\eta_{SF,total}(\omega_{SFG}) = f_{output}(\omega_{SFG})(\int \sqrt{f_{input}(\omega_1)D(\omega_1)f_{input}(\omega-\omega_1)D(\omega_{SFG}-\omega_1)}\, d\omega_1)^2/(\int D(\omega_1)d\omega_1)^2$, where we allow the SFG field generated from different ω_1 and ω_2 to be summed coherently as long as $\omega_1 + \omega_2 = \omega_{SFG}$ and $|\omega_1 - \omega_2| < \Delta\omega$. This requires some level of coherence of the input light at the fundamental wavelengths over a range of frequencies, which is satisfied by the supercontinuum light source. This 'enhancement' is not enabled by the nonlinear material but by the laser source.

The best Comsol simulated SFG results for the disk are shown in Fig. 5(c), where $\Delta\omega = 3.8 \times 10^{13}$ rad · Hz, corresponding to a width of 40 nm around 1400 nm in wavelength. The simulated spectrum reproduces the shape of the experimental results and the total simulated time-averaged power over the wide wavelengths (1300 nm to 1600 nm) is summed to be a time averaged 0.065 nW with an input power of 1.2 mW, agreeing very well with that measured.

13. COMSOL simulation on the excitation and SHG emission of 1 μm disk/Al_2O_3/Ag

The main reason that the E field at the FW is significantly enhanced in the 1 μm disk released on Al_2O_3/Ag is attributed to the excitation of the TM mode, which we refer as the hybrid plasmonic mode in the main texts. To illustrate this effect, we have run FW/SHG simulations on the 1 μm disk/Al_2O_3/Ag, a large disk (diameter > 3 μm) /Al_2O_3/Ag and 1 μm disk/glass an AlGaInP disk. Fig. S13 below shows its electric field amplitude at the FW of three cases up on the excitation of a focused Gaussian beam for comparison. It is clear that the electric field amplitude in the large disk is similar to that of the background excitation field, where we set $|E_b|$=1 V m^{-1}, as well as to that in the disk released on glass, but significantly smaller than that in the 1 μm disk released on Al_2O_3/Ag.

Electric field distribution of 1 μm disk/Al_2O_3/Ag, large disk/ Al_2O_3/Agand 1 μm disk/glass at the FW

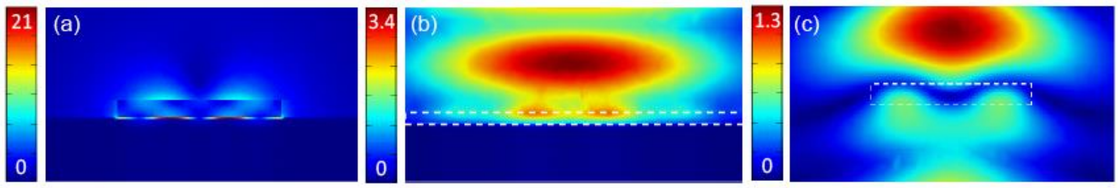

Fig. S13 COMSOL simulation of Electric field |E| at the FW of 1320 nm for the 1 μm disk near metal (a), a large disk near metal (b)and the 1 μm disk on glass (c) when excited by a Gaussian

beam. The E field distribution is plotted on a plane perpendicular to the substrate and across the centre of the disk. The electric field within the semiconductor material near metal is strongly enhanced, about 10 times of the value that obtained on semiconductor released on the glass. The dashed rectangles indicate the location of the semiconductor disk in (b) and (c).

In this hybrid plasmonic TM mode, the E field has a strong out of plane (perpendicular to the substrate) component, as shown in Fig. S14 below. The hybrid TM mode and its out of plane component had been discussed in detail in ref [5-7]. This TM mode is effectively excited in the normal incidence of the pump light when the dimension of the disk is sub-wavelength (1 µm diameter compared to the >1.3 µm FW), where the free-space photons scatter around the perimeter of the disk. The variation of propagation constants Δk can be estimated by the Fourier transformation $\Delta x \Delta k \sim 1$, where Δx can be approximated by the diameter of the disk and Δk is therefore large enough to compensate for the momentum mismatch to excite the TM mode.

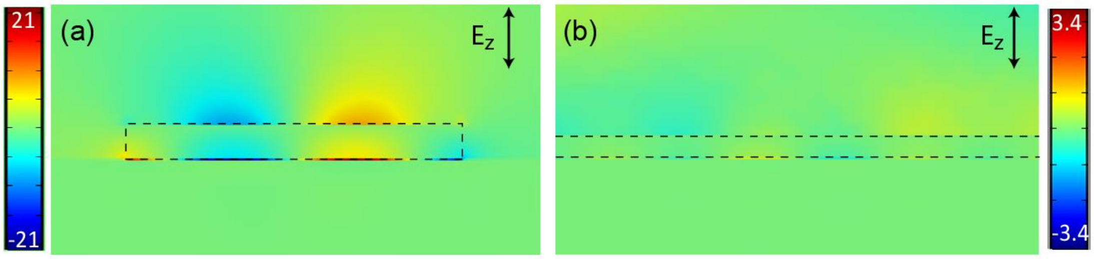

Fig. S14 COMSOL simulation of electric field E_z at the FW of 1320 nm for the 1 µm disk near metal (a) and a large disk near metal (b), as described in Fig. S13.

Most importantly, once the out-of-plane component is excited, E_z component induces dipole moment that is perpendicular to the metal surface, see Fig. S15 (a) below. The image charges induce a dipole moment that is parallel with the original one, the electric field in the semiconductor is therefore strongly enhanced. As a comparison, the effect of E component parallel to the metal substrate is demonstrated in Fig. S15(b), in which the induced dipole is opposite to the original one and therefore no enhancement in the dielectric is induced. The metal particle dimers also exhibit similar polarization dependent field enhancement[8].

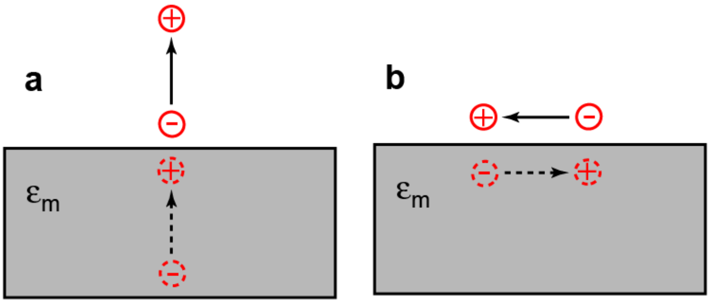

Fig. S15 Diagrams showing that a perpendicular dipole to metal will induce an image dipole parallel to the original one (a) while a parallel dipole to metal will induce an image dipole that is opposite to the original one (b).

SHG simulation of 1 µm disk/Al_2O_3/Ag and far field emission pattern

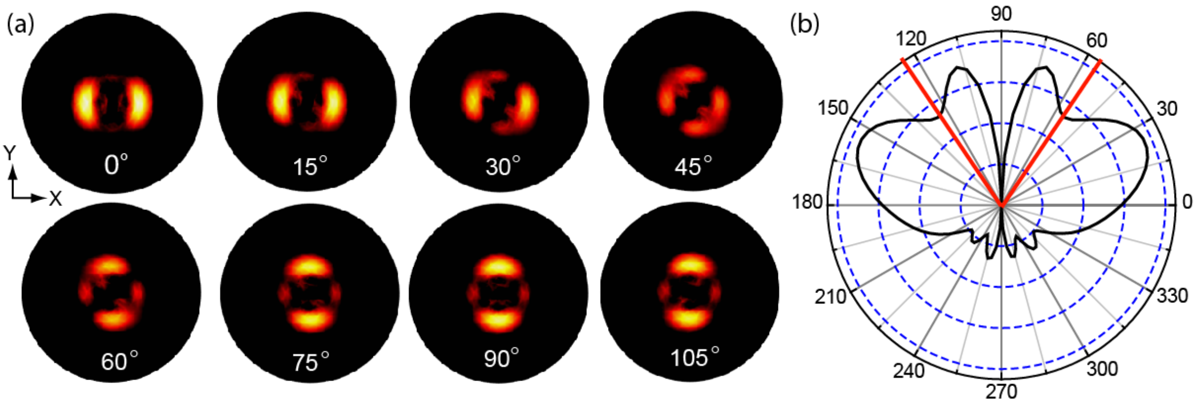

Fig. S16 (a) COMSOL simulation of SHG images $\sqrt{E_{x,SH}^2 + E_{y,SH}^2}$ at 700 nm at various input polarization on a *xy* plane 45 nm above the top surface of the disk. (b) COMSOL simulation on the far field emission pattern of SHG signal at 700 nm with the input polarization along *x* axis. The polar plot is on xz plane with the 0° indicate x axis and 90° indicates z axis. The angles between the two red lines indicate the angles collected by the objective lens used.

References:

1 Davoyan, A. R., Shadrivov, I. V. & Kivshar, Y. S. Quadratic phase matching in nonlinear plasmonic nanoscale waveguides. *Opt Express* **17**, 20063-20068 (2009).

2 Wang, C. *et al.* Second harmonic generation in nano-structured thin-film lithium niobate waveguides. *Opt Express* **25**, 6963-6973 (2017).

3 Ueno, Y., Ricci, V. & Stegeman, G. I. Second-order susceptibility of Ga0.5In0.5P crystals at 1.5 mm and their feasibility for waveguide quasi-phase matching. *J. Opt. Soc. Am. B* **14**, 1428-1436 (1997).

4 Liu, N. *et al.* Lithographically Defined, Room Temperature Low Threshold Subwavelength Red-Emitting Hybrid Plasmonic Lasers. *Nano Lett.* **16**, 7822-7828 (2016).

5 Oulton, R. F., Sorger, V. J., Genov, D. A., Pile, D. F. P. & Zhang, X. A hybrid plasmonic waveguide for sub-wavelength confinement and long-range propagation. *Nat. Photon.* **2**, 495 (2008).

6 Oulton, R. F. *et al.* Plasmon lasers at deep subwavelength scale. *Nature* **461**, 629-632 (2009).

7 Ma, R. M., Oulton, R. F., Sorger, V. J., Bartal, G. & Zhang, X. A. Room-temperature sub-diffraction-limited plasmon laser by total internal reflection. *Nat. Mater.* **10**, 110-113 (2011).

8 Xu, H. X., Bjerneld, E. J., Kall, M. & Borjesson, L. Spectroscopy of single hemoglobin molecules by surface enhanced Raman scattering. *Phys. Rev. Lett.* **83**, 4357-4360 (1999).
